# A qualitative report of the perceptions of the COVID-19 pandemic from collegiate student-athletes

**DOI:** 10.3934/publichealth.2022025

**Published:** 2022-03-15

**Authors:** Dylan C. Rowe, Zachary K. Winkelmann, Shawn M. Arent, Michelle A. Arent, Alexa J. Chandler, Nancy A. Uriegas, Toni M. Torres-McGehee

**Affiliations:** Department of Exercise Science, University of South Carolina, Columbia, SC 29208, USA

**Keywords:** COVID-19, pandemic, lockdown, student-athletes, wellness, support, resources

## Abstract

**Context:**

The COVID-19 pandemic led to an abrupt disruption in access to services and personnel for collegiate student-athletes in the spring and summer of 2020. We sought to identify the effects of this unprecedented change by examining the psychological well-being, changes to normal routines, and return-to-play considerations of current student-athletes in order to guide support for both current and future student-athletes who may face similar situations.

**Methods:**

We utilized a phenomenological approach to interview a purposeful sample of eighteen collegiate student-athletes (7 males, 11 females; mean age = 20 years) from across the United States. The participants were interviewed using a semi-structured interview protocol, which was audio recorded and transcribed verbatim using Zoom. The data were then analyzed and coded by a 3-person team via the consensual qualitative research tradition.

**Results:**

Four domains emerged after data analysis: 1) ambiguity, 2) perspective, 3) bonding and cohesion, and 4) resource utilization. Participants discussed ambiguity in terms of eligibility and participation questions, academic changes, and varying COVID-19 policies. Participants shared a wide range of perspectives, from apprehension at the onset of the pandemic, to excitement when returning to campus and competition. They shared how bonding and team development were affected due to a lack of socialization and that support system dynamics between family, coaches, and teammates were strengthened. When describing resource utilization, participants discussed the use of personnel and supplies to help them adjust to changes in facility and space availability. The identification and utilization of resources enabled them to establish a “new normal” for their academics, workouts, and hobbies during the pandemic.

**Conclusions:**

Collegiate student-athletes realized the seriousness of the pandemic and utilized their resources and support systems to adjust their routines and keep a positive attitude during COVID-19. At the same time, some student-athletes struggled with these changes. Personnel should be aware of these effects to provide care and prevent future negative effects.

## 1. Introduction

The Coronavirus Disease 2019 (COVID-19) is defined as a respiratory illness caused by the novel coronavirus now known as the severe acute respiratory syndrome coronavirus 2 (SARS-CoV-2) [1]. Originating in the central Chinese city of Wuhan [2], this virus rapidly spread across the world, leading the World Health Organization (WHO) to declare it a global pandemic on March 11th, 2020 [3]. The virus is spread primarily through respiratory droplets, which are transmitted to individuals who are in close contact for prolonged periods of time through modes such as coughing, sneezing, and talking [4]. Due to the ease of transmission, the U.S. Centers for Disease Control and Prevention (CDC) released guidelines to aid in the prevention of the spread of the virus, including frequent handwashing, wearing a face covering over the mouth and nose in public settings, cleaning of frequently used surfaces, monitoring of one's health, and avoiding close contact with other individuals, referred to as social distancing, and keeping at least six feet away from others in public settings [5].

As a result of these rising public health concerns, countries all over the globe began to shut down in an attempt to slow the spread. In the United States, the COVID-19 pandemic was declared a National Emergency on March 13th, 2020 [6], leading a majority of states to implement lockdown orders. Between March 1 and May 31, 2020, 42 states and territories issued mandatory stay-at-home orders [7]. Many of these states continued the stay-at-home orders until more information about the virus was released. States then moved to advisory orders, or recommendations to stay-at-home, but it was not mandatory in every state. These stay-at-home orders had a vast impact on schools across the country, as all 50 states closed public schools throughout March, with the majority of colleges and universities following suit and moving to online learning to finish the semester [8]. This shift affected the student-athletes at these colleges and universities, with the National Collegiate Athletic Association (NCAA) making the decision on March 12th, 2020, to cancel the remainder of all winter and spring sports and championships [9].

Before looking at collegiate student-athletes, it is vital to understand the impact of the pandemic on other individuals. Research has indicated that the pandemic caused disruptions to daily routines and an overall decrease in access to healthcare, employment, finances, and mental health [10]. The initial shutdown of states due to COVID-19 caused many business closures, heavily impacting the economy and allowing only essential workers to continue working [11]. While these measures were taken in an effort to protect the general public's health, they had a vast effect across the population, as a study conducted on Americans indicated that those who were experiencing financial hardships due to COVID-19 were more likely to have a higher amount of overall stressors related to the virus [12]. In addition, there have been multiple studies done in the form of qualitative surveys that assess the impact of COVID-19 on measures such as mental health, nutrition, and physical activity for those that have been impacted by stay-at-home orders and quarantine periods [13]–[15]. Results of these surveys show a trend in an overall decrease in physical activity and increase in negative emotions and psychological effects such as depression and stress [13]–[15]. The majority of these published studies come from countries outside the United States, mainly in Europe, China, and Australia, so research is needed to examine whether these effects are applicable to those who live in the United States and also dealt with home confinement and quarantining.

We are especially interested in the effects of the pandemic on student-athletes, as many of them saw their spring 2020 sport seasons cut short due to COVID-19 and had to face a period of time quite unfamiliar for them, as they were forced off campus and had to deal with disruptions to physical training, nutrition, socialization, and academics. Many student-athletes did return to campus in the fall of 2020 following lengthy discussions from the NCAA regarding safety and testing protocols [16]. Most fall NCAA sports (e.g., soccer, volleyball, cross-country) had their seasons and championships postponed to the spring of 2021, except for football, which allowed conferences and individual institutions to make that decision at their discretion [17]. Despite these postponements, many student-athletes were allowed back on campus for practices. As a result, research is needed to understand student-athletes' perceptions of the pandemic both during the stay-at-home time period away from campus, as well as how they were adjusting to returning to school and potentially returning to competition. The purpose of our study was to examine the effects the unprecedented COVID-19 pandemic had on collegiate student-athletes in terms of their psychological well-being, changes to normal routines, and return-to-play considerations.

## 2. Materials and methods

### 2.1. Design and participants

Our study used a qualitative, phenomenological approach to explore the lived experiences of collegiate student-athletes during the COVID-19 pandemic through the use of a semi-structured interview protocol. We recruited a purposive sample of eighteen collegiate student-athletes (7 males, 11 females; mean age = 20 years). Participants were from NCAA Division I (n = 12), Division II (n = 1), Division III (n = 1), and National Association of Intercollegiate Athletics (NAIA) schools (n = 4) across the United States. All student-athletes who were 18 years or older and participating in a NCAA or NAIA sport during the 2020–2021 academic year were eligible to take part in this study. Exclusion criteria included student-athletes that had graduated or were not currently enrolled in school. Table 1 provides the demographic information, including pseudonyms, for the participants.

#### 2.1.1. Ethics approval of research

The University of South Carolina's Institutional Review Board approved this study (Pro00404966). All participant received an interest letter for participation and consented prior to completing the interview.

#### 2.1.2. Instrumentation

An interview protocol was created by the research team, consisting of an introduction, basic demographic questions, and a semi-structured set of questions related to personal experiences during the COVID-19 pandemic and effects on the resumption of the student-athlete's sport season, training and nutrition access, and mental health. Follow-up questions as necessary were asked by the interviewer to ensure the most thorough answers as possible for data collection. The full interview protocol is provided in Table 2.

**Table 1.** Participant demographics.

Pseudonym	Age	Gender	Academic Status	Sport	School Affiliation	Sport Cancelled in Spring or Fall 2020
Adrian	20	M	Junior	Football	NCAA DI	Spring-cancelled Fall-not cancelled
Amy	21	F	Senior	Cross country/track & field	NCAA DI	Spring-cancelled Fall-not cancelled
Camila	19	F	Sophomore	Soccer	NAIA	Spring-cancelled Fall-not cancelled
Charles	18	M	Freshman	Football	NCAA DI	Spring-cancelled (enrolled early) Fall-not cancelled
Debbie	19	F	Sophomore	Volleyball	NAIA	Spring-cancelled Fall-not cancelled
Doug	19	M	Sophomore	Football	NCAA DI	Spring-cancelled Fall-not cancelled
Genevieve	21	F	Senior	Cross country/track & field	NAIA	Spring-cancelled Fall-not cancelled
Gina	22	F	Senior	Lacrosse	NCAA DII	Spring-cancelled Fall-cancelled
Jake	22	M	Graduate	Football	NCAA DI	Spring-cancelled Fall-not cancelled
Kevin	20	M	Junior	Football	NCAA DI	Spring-cancelled Fall-not cancelled
Madeline	22	F	Senior	Volleyball	NCAA DI	Spring-cancelled Fall-cancelled
Raymond	21	M	Senior	Track and field	NCAA DI	Spring-cancelled Fall-cancelled
Rosa	20	F	Sophomore	Track and field	NCAA DIII	Spring-cancelled Fall-cancelled
Sharon	21	F	Junior	Volleyball	NCAA DI	Spring-cancelled Fall-cancelled
Sophia	19	F	Junior	Track and field	NCAA DI	Spring-cancelled Fall-cancelled
Terrance	19	M	Freshman	Basketball	NCAA DI	Spring-in high school Fall-not cancelled
Trudy	22	F	Graduate	Golf	NCAA DI	Spring-cancelled Fall-not cancelled
Vivian	19	F	Junior	Softball	NAIA	Spring-cancelled Fall-cancelled

*Note: M = male, F = female; NCAA = National Collegiate Athletic Association; NAIA = National Association of Intercollegiate Athletics.

**Table 2.** Interview protocol.

Questions
1. Was your sports season canceled in the Spring or Fall 2020? a. If yes, ask questions below. i. How has the COVID-19 pandemic affected you personally as a student-athlete? ii. Tell me about how you reacted when you found out athletics was cancelled for the remainder of the year at your institutions due to COVID-19. iii. Can you share how you are, if at all, preparing to return to your sport. iv. What are your current concerns, if any, with coming back to campus after COVID-19 when thinking about schoolwork, the community, or your life? v. Please describe how you plan on continuing your strength and conditioning programs. vi. Please describe how you plan on continuing your meal planning. 1. Is this different than your previous eating habits? vii. What other activities have you been engaged during COVID-19 without organized sport? b. If no, i. How has the COVID-19 pandemic affected you personally as a student-athlete? ii. What are your current concerns, if any, while on campus during COVID-19 when thinking about schoolwork, the community, or your life? iii. Please describe how you plan on continuing your strength and conditioning programs. iv. Please describe how you plan on continuing your meal planning. 1. Is this different than your previous eating habits? v. Tell us about the safety precautions your institution has in place for student-athletes. 1. Based off these policy and procedures, do you feel safe? 2. Have you or someone you know in your immediate support system (e.g., parents, siblings, grandparents, close friends, significant other, teammates, coach, etc.) been diagnosed with COVID-19? a. If so, how has that affected your experience with the pandemic? b. If no, has this changed your perception of the pandemic? 3. Tell me more about your mental health during COVID-19? a. Have you felt that your school has provided adequate resources to help you deal with any mental health concerns that may have occurred? 4. Tell me more about how you feel about returning-to-sport this year. 5. Is there anything else you would like to share with me relative to sport participation during COVID-19?

#### 2.1.3. Study procedures

Recruitment took place electronically via snowball sampling through the use of an interest letter sent out to various faculty members and athletic trainers from colleges and universities across the United States. These individuals then passed the information along to student-athletes and encouraged them to pass it along to others who may be eligible to participate. The interest letter included the purpose and expected duration of the study, risks and benefits associated with the study, and the research team's contact information to schedule the interviews. We sought to interview a variety of subjects based on sport, grade level, gender, and institutional division level.

Once student-athletes responded, a follow-up email was sent to them to determine a time and date to participate in the interview. All interviews were recorded via a web-conferencing platform (Zoom, San Jose, CA) and were transcribed verbatim. All interviews lasted approximately 30 minutes and were conducted without video, only using the audio aspect for transcription purposes. Following the interview, member checking took place, in which the transcription was sent to the participant to ensure accuracy and allow them to provide any clarifications or updates to their initial responses. The research team conducted interviews until saturation was achieved.

#### 2.1.4. Data analysis

Upon completion of all the interviews, data analysis took place using the consensual qualitative research (CQR) tradition. The CQR tradition allows a team of qualitative coders to review the transcripts for domains and categories and then places frequency counts on the established domains and categories to express the lived experience with a quantitative aspect of how commonly the themes were represented in the participants' transcripts [18]. Our data was analyzed by a 3-member coding team (AAA, BBB, GGG) using a phased approach. These phases consisted of identifying common domains and categories seen in the transcripts, cross-referencing the transcripts by the entire team, meeting to agree on a consensus codebook, and creating the final codebook. Each transcript was then reviewed and cross-analyzed by each member of the coding team to identify the domains and categories based on the codebook. After all analysis was completed, the transcripts were sent to an external auditor to confirm the codes that were identified. The use of the external auditor (EEE) combined with earlier member checking and the triangulation aspect of the coding team established trustworthiness [18]. Finally, each category that emerged was assigned a frequency classification count per the CQR tradition: *general* indicated the category appeared in all or all but one (17 or 18) of the transcripts, *typical* indicated the category appeared in more than 9 but less than 17 of the transcripts, *variant* indicated the category appeared in between 4 and 9 of the transcripts, and *rare* indicated the category appeared in 0 to 3 of the cases [18].

## 3. Results

A total of 18 individuals responded to our invitation to participate in the study. All 18 met our inclusion criteria as they were currently enrolled in school and were older than 18 years of age. As a result, none of the respondents were denied enrollment and all interviews were completed as scheduled. Recruitment ended following the 18^th^ interview, as the research team determined that saturation had been reached. Following data analysis of these 18 transcripts, four main domains emerged to describe the student-athletes' experiences during the COVID-19 pandemic: (1) ambiguity, (2) perspective, (3) bonding and cohesion, and (4) resource utilization. Each domain consisted of specific categories to further illustrate the lived experiences of the student-athletes that were interviewed. Table 3 provides the frequency counts for each of these categories. Additional quotes supported in the interview excerpts are provided per domain in Table 4 (Supplemental Data).

**Table 3.** Frequency counts.

Domain and Category		Frequency	CQR Assigned Value
Ambiguity		
Eligibility and Participation		17/18	General
Academics and Classes		8/18	Variant
COVID Policies and Procedures		16/18	Typical
Perspective		
Apprehensive		17/18	General
“COVID is Real”		18/18	General
Motivation & Lack of Motivation		15/18	Typical
Policy Adherence		18/18	General
Feeling Safe		14/18	Typical
Excitement		16/18	Typical
Bonding and Cohesion		
Lack of Socialization		13/18	Typical
Team Development		12/18	Typical
Support System Dynamics		16/18	Typical
Resource Utilization		
Personnel		17/18	General
Timing and Facility Space		16/18	Typical
New Norm		18/18	General

### 3.1. Ambiguity

The domain of ambiguity was established to describe the sensation of uncertainty, disappointment, and confusion student-athletes felt surrounding the COVID-19 pandemic. The categories covered in this domain were eligibility and participation, academics and classes, and COVID-19 policies and procedures.

#### 3.1.1. Eligibility and participation

The student-athletes initially described a feeling of ambiguity through concerns regarding their participation status and eligibility. Many of these athletes had their sports seasons cut short in the spring of 2020 with no ideas of what the upcoming weeks or months would look like. Jake explained this feeling by stating:

“We got a text message from the head coach saying that we cannot come back to school, and that spring ball is going to be cancelled. Everything was kind of in the air for about a week and a half, and then all the quarantine and lockdown started and that is when I am thinking ‘Is school cancelled for the rest of the semester? Obviously, we are not going to have spring ball, like what is the season going to look like? Are we going to play?’ I think around that time all the spring sports had gotten cancelled, as well. So, the eligibility questions were starting to rise for me.”

#### 3.1.2. Academics and classes

Based on these experiences, student-athletes expressed concerns and had multiple questions regarding their athletic status and when they would be able to return after COVID-19 had ended their spring seasons. Academically, once the initial shutdowns and quarantines had ended, they also expressed ambiguity when returning to school and attending classes. When asked about some of his concerns when returning back to campus after the break, Charles responded:

“Definitely one of my main concerns was schoolwork, because these were trial-and-error types of situations. Teachers and professors were trying stuff they have never tried before. It could work out, it could not, and sometimes it did not work out. Sometimes it did have a negative impact. Classes were harder just because of the nature of the situation.”

#### 3.1.3. COVID-19 policies and procedures

Finally, in addition to these academic concerns, ambiguity was shown through university and teamwide COVID-19 policies and procedures, which were described by the participants as being vague and constantly changing. There was specifically concern surrounding how the general student body would be mixing in with the student-athlete population and if there would be any policies to combat this. Amy talked about this concern by stating:

“We were framing it as our biggest threat to the season not happening was the student population at large, because that population is so much bigger than the student-athlete population is. We knew if the school got it, especially in the beginning when we were concerned, everyone was going to get sent home. Then where would that leave us in terms of having a season? That was our biggest concern, because if there was a big outbreak on campus, it was going to filter over to athletics.”

### 3.2. Perspective

The next domain to emerge following the interviews was perspective. Student-athletes described a wide-ranging change in perspective due to the pandemic, which was shown in 7 different categories to explain the process of this perspective shift. These categories were apprehensive, a feeling that “COVID is real”, motivation, lack of motivation, policy adherence, feeling safe, and excitement.

#### 3.2.1. Apprehensive

The student-athletes interviewed described a very apprehensive feeling when they first heard about COVID-19 and had their sports seasons cut short. Adrian explained his feelings at the onset of the pandemic by stating, “I will say my reaction back then was probably fearful. I was probably more scared than anything for my family and my life.” There were also apprehensive feelings from more of a sport perspective, as Jake described:

“I was pretty upset at first. When you look at it, I guess from a team perspective, we have a really good football team that can win a lot of football games, have a shot to win a championship, so that was disappointing.”

#### 3.2.2. COVID is real

Following these initial apprehensive and disappointed feelings, the student-athletes began to have a shift in perspective as they realized that the pandemic was a real thing and that it was affecting people in a very serious way. Raymond explained the situation well, especially considering he had teammates that were affected by COVID-19, as he said:

“I think seeing them get it made me more cautious because I realized college kids can get it. Not that I didn't think that before, but it is more real when it hits closer to home. I realized it was serious because I heard stories of older people getting it. I realized even if I did not necessarily feel bad from catching it, giving it to other people concerned me, because they could not necessarily fight off the virus. So, it made it more serious, but I knew this was not something to mess around with, and you should be as safe as possible.”

#### 3.2.3. Motivation

Once the student-athletes were forced away from campus due to lockdowns and quarantine guidelines, some varying perspectives began to arise. Some athletes did a good job focusing on aspects of their life they could control, while others did not. Kevin described his positive thought process during the pandemic by saying:

“I was making sure I was doing every single workout they sent us plus more. I wanted to make sure I was in shape because I could not really tell if I was fully getting the amount of work I needed. And I did not know how I would be returning if I was going to be out of shape or not. I definitely upped my training once we finally got a date. We had a little over two weeks' notice- and once we finally got that date, I definitely turned it up even more with my training to make sure I was ready to be back.”

#### 3.2.4. Lack of motivation

On the other hand, some athletes expressed negative emotions while they were away from school and sports. Sophia explained this situation and how she struggled without the resources of being at school by saying:

“I remember the biggest challenge for me was motivation and not having the equipment or not having my teammates around me. I remember that making it a lot harder to get myself to do the workouts or everything I needed to do.”

In addition to this lack of physical motivation, some athletes described the mental effect of not being able to play during the pandemic, which affected their motivation just as much. Madeline described her feelings, stating:

“Being a college student-athlete during the pandemic, looking back and I guess forward, it definitely feels like an honor. I say it in the sense we were able to do a lot of things people were still fighting to do in the public eye. Sometimes student-athletes get a bad rap. People tell you we chose this life, and we did, but still to have that one thing taken from you, it is difficult. Everybody comes from different backgrounds, we faced different things our lives, and volleyball has always been an escape for me. To have that one thing taken away to the point where you can't even go to a gym if you try, it is overwhelming.”

#### 3.2.5. Policy adherence

Following the initial shutdowns, athletes slowly started to return to campus in the summer and fall of 2020. Their perspectives continued to change as they realized that they needed to follow all necessary COVID-19 policies in order to be able to play their sport as quickly and safely as possible. Jake explained how his team approached the situation, describing:

“For the most part I think if you want to be able to play football, then you have to do these things. So the guys that pushed back against it quickly realized if you want to work out, or if you want to play football, then you have to listen and you have to take these tests, to take your temperature, you have to social distance and wash your hands.”

#### 3.2.6. Feeling safe

With all of the policies the student-athletes were adhering to, the vast majority of the participants held a perspective of feeling safe on campus, especially with the resources they were provided. The majority of the student-athletes interviewed were tested for COVID-19 regularly, which they said made them feel better. Kevin explained this thought process:

“I was never too worried about it in the first place. I felt a lot better being tested all the time, so I would know if I had it, especially if I was around other people. If I saw my grandparents, I knew I would not give it to them. I felt a lot better knowing we were always being tested and I would not have to question if I had it or not.”

#### 3.2.7. Excitement

Following all of these changes and perspective shifts, the overwhelming majority of the student-athletes expressed excitement for returning to campus and sports. After the long period of quarantine and stay-at-home orders, Rosa described her emotions on returning to school:

“I cannot even tell you how excited or happy I am. I mean when I think about it, it just makes me smile and happy. I miss my teammates. I miss my friends. I miss walking to class. I am sick of getting out of my bed, walking down a flight of stairs, and not moving until I get out to run.”

### 3.3. Bonding and cohesion

The third domain to surface after data analysis was bonding and cohesion, as student-athletes found themselves forced away from campus, altering their relationships both at school and home. This domain was shown through the categories of lack of socialization, team development, and support system dynamics.

#### 3.3.1. Lack of socialization

Socially, many student-athletes found themselves struggling with changes due to COVID-19 that affected how they were able to spend time with friends and teammates. Even being back on campus, the student-athletes reported having less socialization than usual, partly due to closures of school facilities. Raymond explained his experiences by saying:

“Those first three years I had been around people the whole time. So having to tighten it a little bit was a tough transition. You cannot really see people outside of your sport, outside of your crew, and you could not really meet new people. Having [the dining hall] shut down was also weird because I am used to eating dinner there and socializing. So that was a big part that was cut out.”

#### 3.3.2. Team development

The participants also described how team dynamics were greatly affected due to the pandemic and the restrictions that came with it, both in negative and positive ways. Negatively, certain protocols affected the way the team was able to bond and connect with one another. Gina explained this by saying:

“We are not allowed to use our locker room this year, which has changed a lot of the team dynamic. I would say that is honestly a big part of our social aspect, and gets us ready for practice, and then we are not even allowed to bring it in close for a huddle, too.”

However, some of the athletes described how their team development was improved, since their teammates were some of the only people that they could see during the pandemic amid all of the strict guidelines. Some even lived with teammates, which helped strengthen their bond, as Kevin discussed:

“I live with six guys and they all, or almost all, play football. Just constantly around them, constantly having those guys that going through the same things as you, it is easy to stick together.”

#### 3.3.3. Support system dynamics

With all of the uncertainty and changes going on, the overwhelming majority of the student-athletes interviewed discussed how they had to rely on their support system to help them get through the pandemic. This included individuals such as family members, friends, teammates, and coaches. Vivian shared how having a good support system helped her, especially with her mental health, when she said:

“I still had days where I was a little depressed, just because I was like, ‘Is this ever going to end? Are we ever going to get back to normal?’ But working and being around people and having human contact really did help during the pandemic. Then once we came back to school and everybody was here, I was seeing everybody, and we were all hanging out, I was a lot better just being around people who we all have something in common, we all know each other, and we can all have a good time and still be safe. So I was depressed for a few months, but once we came back to school and everyone was back on campus, I felt a lot better.”

### 3.4. Resource utilization

The final domain to emerge after the interviews was resource utilization. After being forced away from campus and sports, student-athletes found themselves lacking their usual resources and had to adjust accordingly. The categories from this domain were personnel, timing and facility space, and new norm.

#### 3.4.1. Personnel

One way student-athletes navigated their way through the pandemic was by using the personnel from their schools as a key resource. This included strength and conditioning coaches that were proactive in making sure athletes were staying healthy and in-shape despite not being at school and having their usual workout equipment. Charles described the effect of his strength coach by saying:

“My strength coach did send us a [workout plan] to do over the break. But it was not too rigorous, because he tried to accommodate for people who did not have access to weights. I did his workouts and also did my workouts with lifting weights, working out with people at the field from my high school, and just trying to get bigger, faster, stronger.”

Other individuals that the student-athletes relied on during the pandemic were mental health personnel provided through the school. Vivian described the resources that her school provided, stating:

“Our school offers counseling services to any student for free. I never went personally, just because I am not one to talk to someone I do not know, but I do know a few teammates who went to her and spoke to her and the school actually had a COVID-19 relief counseling club, so any student-athlete could join that group on Zoom and just talk about COVID-19 and talk about things to help your mental health.”

#### 3.4.2. Timing and facility space

Student-athletes found that changes in facilities due to COVID-19 also forced them to utilize different resources, as they had to adjust their workout schedules and dietary preferences. When returning back to campus, many of the athletes also shared concerns about how workouts would change, since many of their facilities were altered due to the new COVID-19 policies and guidelines. Rosa expressed this feeling, stating:

“I am concerned if training is going to be as good as it was before. I know we are not going to have as big of time blocks. Before we had three hour practices every day. But because they need to fit in so many sports into our facilities, that is just not a possibility because you cannot have as many people in the facility at the same time. So, I am concerned about the quality of our workouts.”

These workout changes forced the athletes to modify their daily routines and use resources in different ways. In addition to these changes, dining facilities were also greatly impacted, which had similar effects on routines. Sophia explained:

“Our dining situation this semester is different. We actually have an athletic dining hall that we usually have buffet style: you go in, you get your food, and you sit down. This year, when you walk in, you swipe your card, and they have a little menu of what they have that day. They have everything in individual boxes, you tell them what you want, they put it all in the paper bag, and you leave. So it's a lot different.”

#### 3.4.3. New norm

Lastly, due to all of these changes, student-athletes described the identification and utilization of resources which enabled them to establish a “new normal” for their academics, workouts, and hobbies during the pandemic. Being back home, some athletes did not have access to gyms or workout equipment, which Sophia described:

“I would just do bodyweight workouts in my basement area. But the main thing I remember from being in quarantine was I just was not doing enough, and I was not doing all the workouts and it was not exactly how the workouts were supposed to be done because I was inside. I didn't have a track. I remember I was doing a version of stadiums in my house, by just running up the two floors of stairs. So, I was thinking creative in what I was doing.”

Finally, with all of the free time being away from campus and sports, many of the athletes picked up hobbies as a way to spend their time. Vivian explained her experience, saying:

“I have always loved art and I actually used to work at an art studio before my freshman year of college, so I picked up a little bit of that because I was not able to do it in college, since I did not always have time. I used that as my stress relief and just having something to do because I could not do softball. Then it got to the point where I was like ‘Well I need a job,’ so I went job hunting. I found a job to do over the summer that I continued in the fall up until February, so that is all I did.”

## 4. Discussion

As stated previously, the COVID-19 pandemic was an unprecedented event that drastically changed the daily lives of college student-athletes. All participants in this study had their respective spring seasons of 2020 cancelled. Therefore, they were forced to manage the abrupt change, which greatly affected their resources for both academics and athletics in the spring and summer of 2020. The majority of student-athletes then returned to campus for the fall semester. Describing how these student-athletes were able to navigate through the stay-at-home period with limited resources, as well as their differing experiences once back at school, is crucial to assist coaches and support staff in the event of future extended breaks from supervised training. The participants in this study described both positive and negative outcomes on health, wellness, and performance in regard to the COVID-19 pandemic, which can be used to guide this type of future research.

### 4.1. Ambiguity

Due to the unique nature of the COVID-19 pandemic, it is no surprise ambiguity was a main theme to emerge from the experiences of the participants. As this was the first time all sports have been completely interrupted since World War II [19], there was no true precedent for how to approach this situation, leading to various questions from the student-athletes involved. Athletic eligibility and participation questions were some of the common concerns that arose, as the student-athletes were essentially forced off of campus with no idea of when they would be able to return and compete again.

Another topic that created a lot of concern and uncertainty was the consequence of a positive COVID-19 test on the physical performance of athletes. Early research indicated COVID-19 infections were associated with cardiac involvement which led to questions about whether positive student-athletes should undergo cardiac testing before being allowed to return to their sport [20]. Despite not being mentioned by our participants, this was a large discussion point for many colleges and universities in terms of return-to-play policies. Following additional research specifically looking at collegiate student-athletes, it was determined there was a low prevalence of cardiac involvement and a lower risk of adverse cardiac events for student-athletes who tested positive for COVID-19 [21]. Therefore, policymakers could feel safe allowing student-athletes to return to physical activity following a positive test, which helped to ease these concerns.

In addition, academics were also up in the air. Almost all college students in the U.S., regardless of if they were an athlete or not, had to finish their spring 2020 semesters online [8], leading to concerns from our participants about distance learning and how that would affect them. These concerns are in line with research that explored issues associated with online learning at the onset of the pandemic, such as how to deliver end-of-semester assessments, complete lab and practical work, and assist students who may not have internet access outside of campus [22]. The participants in our study also shared concerns with academics once they were back at school in the fall of 2020, as there was uncertainty on whether classes could be held in-person or whether they would continue to be online.

Lastly, participants described ambiguity in terms of the COVID-19 policies and procedures in place at their institutions once they were back on campus. A large concern was the mixture of the general student body with the athletic population. Some participants were worried that an outbreak with the student population could spread to the athletes and affect their sports seasons. Others shared that the policies on campus were not followed very seriously, leading to fear and untrustworthiness. First-hand accounts like this can guide future policy makers and administration to ensure that strict, clear protocols are in place.

### 4.2. Perspective

The participants described a plethora of different emotions related to COVID-19, leading to changes in their perspectives as the pandemic played out. Initially, the majority of them shared a feeling of apprehension related to fear of the virus and anxiety on how it would affect them and their daily lives. These emotions align with previous research demonstrating similar negative impacts on mental health for the general public at the beginning of the pandemic [15],[23]. The same effects have also been recognized in surveys taken by college student-athletes, with reports of increased stress, fear, and general concerns about the future being the most prevalent [24],[25]. As the pandemic continued onward, participants came to a realization COVID-19 is real and should be taken seriously. This finding was unique to our research, illustrating how the mindset of college student-athletes evolved as more information came out and people close to them began to contract the virus. These specific personal effects guided individuals' thought processes and emotions throughout the pandemic. The next aspect of the perspective shift, in which participants described motivation or lack of motivation while following the stay-at-home orders, exemplified these personal effects. Previous research has examined the effects of the initial lockdown on motivation, with the majority of results revealing lack of motivation, overall mood, and a lack of physical activity [24],[26]–[28]. Many of our participants shared similar outcomes, although there were a few that explained how they were positively motivated by the free time, further indicating how the pandemic affected certain individuals differently. Our participants were all intrinsically motivated to stay in shape, so future research is needed to examine other possible reasons for motivation.

Another unique finding was that some of the lack of motivation shared by the participants was due to the general public's perception on student-athletes playing during a public health crisis. Some of the student-athletes explained how they felt targeted for playing during COVID-19, but it was necessary for their own mental health. This disconnect between the public and student-athletes is something that can be explored in future research. Next, as student-athletes began to return to campus in the fall of 2020, they shared how their perspective further shifted as they began to compete again. Adhering to COVID-19 policies became a priority for the participants as they longed to be able to play sports again. This finding was noticeable, as around this time, research identified a feeling of “pandemic fatigue” amongst the general public, with global reports of decreased adherence to policies such as physical distancing guidelines [29]. The extrinsic goal of participating in sports allowed our participants to avoid this phenomenon. As a result, the majority of the participants shared they did feel safe being back on campus. Having strict policies, accessible COVID-19 testing, and individuals with the same mindset as them to hold them accountable were the main reasons that these student-athletes felt safe on campus.

Finally, as sports and competitions began to return, participants shared an overwhelming feeling of excitement to be able to play again. Actually, being back on campus with teammates and being able to compete rather than just train were some of the main reasons for this excitement. These findings can be associated with previous research indicating physical activity and athletic identity helped many student-athletes maintain positive mental health during COVID-19 [30],[31]. By being back on campus, these student-athletes were able to regain their athletic identity and compete for the first time in months, which led to their excitement directly related to the return of sports.

### 4.3. Bonding and cohesion

The findings suggested being away from campus affected participants' relationships with family, friends, teammates, coaches, and other members of their support systems in both positive and negative ways. Lack of socialization was a main topic brought up by our participations. This may be because of the initial stay-at-home orders and later, the stringent COVID-19 social distancing policies back on campus. Previous research identified negative effects of isolation on athletes' mental health, leading to symptoms such as increased depression, anxiety, and stress [28],[32],[33]. Our research displayed similar conclusions, with student-athletes also exhibiting these effects back at school in the fall of 2020 as they dealt with policies such as closed dining hall facilities and COVID-19 rules that prohibited the use of locker rooms and other team common areas. Student-athletes also realized how much these experiences had strengthened their team bonding and socialization in the past, and it greatly affected them once they had it taken away from them. In addition, the freshmen participants in this study struggled as they were completely new to their schools and found themselves unable to interact much with their own teammates. Prior research identified proactive social behaviors are important for freshmen transitioning into college [14], and our research aligned with this concept, as our freshmen student-athletes also struggled due to their lack of social interaction.

There were also some benefits that came about due to the pandemic. Participants described how relationships with other individuals were strengthened due to living through this shared experience. In the initial stay-at-home phase of the pandemic, the student-athletes shared how family members and friends made a large difference. Some explained school personnel helped them the most initially by constantly checking in and making sure they were okay. Once everyone was able to return to campus and interact following the initial lockdown period, it was the relationships with teammates that were especially strengthened. After being away for so long, many participants realized how much they valued human interaction and the social support their team brought. Our results aligned with previous research demonstrating how student-athletes have higher levels of self-esteem and social connectedness than non-athletes [34], due in part to these relationships with teammates. Participants emphasized how these support system dynamics truly helped them get through the pandemic. Similarly, collegiate student-athletes have demonstrated that higher levels of social support is correlated with fewer depressive symptoms [35], which is the likely reason that our participants felt this way. Most of these findings emphasized social support through teammates and friends, but school personnel can also provide necessary support. In college student-athletes who suffered an injury, higher levels of social support from athletic trainers and strength coaches were associated with lower levels of depression and anxiety during rehabilitation and return-to-play [36],[37]. Long-term injuries can result in similar feelings of isolation that the pandemic caused, so these findings further emphasize the necessity of strong support systems. We suggest stakeholders use this information to guide future team and schoolwide protocols to ensure efforts are made to allow for socialization, whether this be virtually or in-person when applicable.

### 4.4. Resource utilization

By being forced away from campus for an extended period of time, student-athletes were forced to adjust their resources in order to continue normal strength and conditioning training. Many student-athletes shared they had programs sent to them from their strength and conditioning coaches that allowed them to continue their training regimens away from school. Some student-athletes had workout equipment available to them at home, while others had to be creative and mainly do bodyweight (calisthenic) workouts. Similarly, Chandler and colleagues surveyed collegiate student-athletes during the pandemic and found 38.7% (137/354) of them could fully perform their training programs away from school due to a lack of appropriate training equipment [24]. This is likely due to the fact the pandemic occurred so suddenly and did not allow for student-athletes or personnel to plan ahead. In future situations, personnel should consider each individuals' resources and provide options to guarantee training can still occur. Researchers have emphasized the importance of continued training during extended breaks in order to prevent the effects of detraining, which includes decreased speed, power, and aerobic capacity [38],[39]. Therefore, in future breaks from supervised training, like the pandemic, considerable efforts should be made to ensure student-athletes are able to continue their routines wherever they may be and avoid these negative effects.

In addition to strength and conditioning personnel, student-athletes shared they used mental health personnel as a resource away from school, primarily through video-based conferencing (telehealth). Using online resources as a means for mental health treatment has been gaining popularity since the early 2000s [40], but grew even more during COVID-19, mainly out of necessity. Telehealth has been shown to be an easy and effective way to provide mental health support for those that may need it [41]. Identifying and communicating telehealth as a primary resource for student-athletes is key in future situations. Finally, participants shared the pandemic allowed them to pick up new hobbies and routines with all their free time away from sports. These hobbies were beneficial as they were often activities the student-athlete found enjoyable and helped ease their stress levels. Previous research indicated elite athletes have used cognitive and behavioral coping strategies to deal with stress related to the pandemic [42], but our findings are some of the first to demonstrate the beneficial effects of extracurricular activities. Continuing to engage in similar activities with the return of sports can help student-athletes maintain optimistic attitudes and ensure their identity is not solely in their sport [43]. Overall, finding a way to adapt to this “new normal” demonstrated positive outcomes for the participants and allowed for the majority of them to ease back into sports with very little issues. It may be beneficial for colleges and universities to plan ahead and invest in resources that can be utilized outside of campus to assist student-athletes who undergo similar situations in the future.

### 4.5. Limitations and future research

Our study is not without limitations. Participants were recruited using both purposeful and snowball sampling, which could have resulted in selection bias. While an effort was made to recruit a wide variety of athletes, the findings may not be generalizable to all collegiate student-athletes. In addition, the interviews began in the winter of 2020 and went through the late spring of 2021, so some of the athletes were asked to recall events that had occurred over a year earlier.

Future research should continue to explore student-athletes' physical activity and nutrition patterns to identify the impact of the pandemic on these health behaviors, specifically during the stay-at-home period compared to their return to campus. A previous study by Christensen et al. [44] examined specific eating habits in relation to mental health status during the pandemic by utilizing the Diet History Questionnaire (DHQ-III). We could have incorporated a reporting tool like this into our study to examine the relationship of nutrition to our areas of focus. Our participants reported no drastic changes to their nutrition status, but it would have been beneficial to have used a specific outcome measure. Finally, additional follow-up research should also be conducted to examine the continued and lasting effects of COVID-19 on student-athletes as colleges and universities continue to compete during the pandemic.

## 5. Conclusions

The COVID-19 pandemic was unique in that student-athletes and other athletics personnel had never experienced this type of abrupt shutdown in their lifetime. Therefore, they did not have the opportunity to plan for this drastic change. Amidst uncertainty, collegiate student-athletes responded in various ways as they realized the seriousness of the situation and the pandemic began to affect their daily lives and routines. Some student-athletes were able to focus on aspects within their control and utilize various resources to keep a positive attitude towards the “new normal” of the 2020 sports year. Others perceived a more negative effect and struggled to adjust. Overall, having a good support system and adequate resources seemed to help the student-athletes the most and should be made a priority in future similar events. We encourage proactive planning by stakeholders to ensure that the lessons learned from the pandemic are applied when providing care for student-athletes away from campus and to prevent any similar negative effects from occurring.
