# Supplementary material for: A qualitative report of the perceptions of the COVID-19 pandemic from collegiate student-athletes
Source: AIMS Public Health. 2022 Mar 15;9(2):357–77. doi: 10.3934/publichealth.2022025 (PMC9114786; doi:10.3934/publichealth.2022025)
Supplement: Supplementary file 1 [file publichealth-09-02-025-s001.pdf]

AIMS Public Health

http://www.aimspress.com/journal/aimsph

AIMS Public Health, 9(2): 357–377.
DOI: 10.3934/publichealth.2022025
Received: 30 December 2021
Revised: 22 February 2022
Accepted: 02 March 2022
Published: 15 March 2022

Research article

A qualitative report of the perceptions of the COVID-19 pandemic from collegiate student-athletes

Dylan C. Rowe, Zachary K. Winkelmann, Shawn M. Arent, Michelle A. Arent, Alexa J. Chandler, Nancy A. Uriegas and Toni M. Torres-McGehee*

Department of Exercise Science, University of South Carolina, Columbia, SC 29208, USA

* **Correspondence:** Email: torresmc@mailbox.sc.edu; Tel: +18037770636.

Supplementary data

Table 4. Supplemental quotes by domain and category.

**Ambiguity Domain**	
Eligibility and Participation	• It was upsetting when we found out athletics were first canceled because everything was so step-by-step. We just thought our season is going to be postponed, that is disappointing, and then it was cancelled. At least we will be back for the next semester. Then next semester is cancelled, and the next season is cancelled. So, you hold out hope at the beginning, but it seems to be never ending, it is pretty saddening. You do not know when the next time you will be able to compete is. I have not competed in almost a year now. – Rosa<br>• A lot of questions were about eligibility, and I was glad our eligibility will not be counted for the spring season, and we can add guys back in the fall. Only main issue is if we get hurt, it is a lot of football in the spring and the fall for injuries. Probably most likely going to be out for both seasons, which is the worst part of it, but that is football. – Charles
Academics and Classes	• I have never been the one to break protocol and get sick, but it happens, and in those two weeks, I obviously could not go to class.

	Our professors were not recording things anymore because we were all there, so I had to Facetime into class, I could not go work in my lab. That was the biggest impact for me, was being quarantined and missing class. – Madeline
COVID-19 Policies and Procedures	• When we trained in the athletic room for workouts with my team or any of the other teams, no one took it seriously because you take off your mask when you are working out, but it just did not make sense. You are working out, but then you take off your mask and then you are also touching all the equipment, and no one really cleans them. – Trudy<br>• Sometimes we would not even wear [our masks] in the dugout I will be honest, because we were hot, we were tired, and it was just too much. But if coach said “Hey, please put your mask on,” we would do it, but a lot of people as soon as they stepped foot out onto the field, they took their mask off because they were just kind of sick of it, and it was really hot sometimes. – Vivian
**Perspective Domain**	
Apprehensive	• My mental health stayed pretty positive, other than the fact there was so much anxiety and fear for the virus. I would just say that was probably the biggest burden on my mental health. – Sophia
“COVID is real”	• COVID is a real thing. People are dying from it. It is very dangerous. It needs to be taken really seriously. So once you realize that, for example, my grandma is in a nursing home, and she still has not been able to get out. We have not been able to see her at all. I have not seen her in months. When you put that in perspective, you sit with it, and it is what it is, you have to move with it. – Jake<br>• To be honest, I did not understand a lot about COVID. And when our most important tournament got cancelled (NCAA tournament) it was then I realized that this is a real deal. – Terrance<br>• You always hear about things, but you never think it is going to happen to you. So, for my school and all the schools around me to cancel the rest of the season for athletes and send everyone home, including the people who are not athletes, it felt very real. It felt like it was a wakeup call. – Debbie
Motivation	• A lot of guys can do two things. You are going to either get better as an athlete or you can deteriorate and get worse. I did not take it as a way for me to get worse. I took it as a way for me to get better because I knew a lot of people would sit back and say, “Hey, we are in quarantine, or we have to be isolated. We are stuck in our house or stuck in our apartment, and we cannot do much. So, we are just going to be lazy and not take advantage of free time.” That was not the case with me. I worked out two to three times a day. I stayed in my playbook. – Jake<br>• Ultimately it was a good thing for me, knowing you went through something, you are still going through something, and trying to

	succeed at something while not a lot of people before you and after you might not be able to go through, might not have that experience. It made me mentally tougher. – Charles
Lack of Motivation	• A lot of people did not want it to happen, and some people were saying "If there are people dying out there, why are you prioritizing this and competing, like that is not important, and you should not be risking other's lives just to compete." There was a little bit of dialogue surrounding athletics at the beginning and even throughout. I do not think people realized how much not being able to compete affects an athlete's mental health, and how if you cannot compete and you have built your life, or half your life, 10 years, around a sport and then suddenly it is not available to you, I do not think people realized what it does to you. – Genevieve<br>• People do not give us enough credit for doing what we do throughout the year, and throughout the pandemic. From an outsider's perspective it looks like "Oh, you just want to play your sport blah blah blah." But I know our football team, they had a season in the fall, and it was just brutal. Obviously, these kids had just as much time as we did to prepare for the season, which was none. We had minimal resources until things opened up, and you see on social media these grown men just bashing these kids. I think there is a lack of understanding both from the general public, from a sports fan aspect, and the student population and professors. They do not understand that it has been really hard mentally, physically and we are all burnt out. We have been doing it for a long time because we have to practice like we are playing tomorrow, every single day. – Sharon
Policy Adherence	• I took it personally. I made sure I was where I was supposed to be and I did everything I was supposed to do, washing my hands, and making sure I stay 6 feet, keeping my mask up. Once you make that decision for yourself, you can feel as safe as you are going to feel anyway because it is kind of inevitable. – Adrian<br>• I knew I would have to test like two to three times a week, which would just be annoying to get stuff shoved up my nose, but more or less I was not worried. I just have to do a little more stuff that I usually would to be able to play football and go to school, but you know I did not really mind, I was glad to be back. – Doug<br>• It was a really big struggle preparing to go into college. We had to do all these extra things that we normally would not have to do. We had to get COVID tests done to make sure all of us were fine; and then, when I had gotten to my college, we were given masks, given hand sanitizers and thermometers, just so everybody has them on our campus, so that we were all taking the right precautions. – Camila
Feeling Safe	• I felt I was probably safer up on campus than I would be back home. Because people were taking a lot more seriously at the campus. If you

	were not walking around with a mask, someone was going to call you out for it. If you were not wearing a wristband you would not be let into position meetings or weightlifting sessions, so if you did not do what you needed to do, then you were not going to be able to participate in any team activity. – Jake<br>• Us being a small private college, we did not have as many people as most colleges or universities did. So, taking these precautions and having these rules in place to where you have to wear your mask, if you are feeling sick, you cannot show up to a class if you have an in-person class, things like that. Our college really made it to where we were able to feel safe. – Camila
Excitement	• I was so excited; I could not believe it was actually happening. I was so dead set if I was not coming back in the fall, there was going to be no reason for me to come back in the spring. I love volleyball do not get me wrong, and I want to get better at it and that is what the spring season does for you, but I want my matches, I want to play. – Debbie<br>• Volleyball has always been an escape for me and to have that one thing taken away to the point where you cannot even go to a gym if you try, it is overwhelming. As we started to get all that back and we started to just get on the court with each other, I had never seen such positive energy from my team before, because we were just so excited to see each other again. – Madeline<br>• I was incredibly excited to return to sports. I had a mix of other emotions: "Is it going to be the same, am I still going to enjoy it, am I going to be able to compete at the same caliber," and those went away fast when I realized the competition is the same, it is just the stuff in-between that is different. – Genevieve
**Bonding and Cohesion Domain**	
Lack of Socialization	• My whole mindset changed. I was depressed being in [isolation] alone, obviously, it was really hard. Especially for myself and my whole team, we are a very social team, and we are always around each other and always hanging out, and there was quite a few of us that had to quarantine by ourselves which was definitely tough. We still had Facetime and stuff like that, but it was just not the same as being with people. – Gina<br>• I did not know anybody that played at the school that I play at. I was a freshman; I was new to all of this. We have to do these workouts on our own, and then we get put into college, and then we meet all these new people, and we cannot even interact fully because of COVID. – Camila
Team Development	• I still get like the basic stuff, get my practices, get my workouts done and everything, so I would just say more physical interaction. I mean I only had physical interaction with my teammates, but outside of our group, I did not have as much. – Terrance

Support System Dynamics	• Our coaching staff, they did a great job of communicating over the quarantine with us, making sure we were still good, doing what we were supposed to do. I feel like they are like family. If you ever need anybody to talk to, you can always call on your coaches, your position coach, head coach, he is the same way. And our whole entire staff, our training staff down to our strength and conditioning staff, I feel like everybody is there with us. You are not dealing with it alone. – Adrian<br>• I was very fortunate to live in a house where I had a loving support system and I had things to do, places to walk around safely. A lot of people went home to being in a home alone or being in a home where they were not loved and supported as much as I was. I can easily see if I went back to a lonely place, after being stripped of everything that I felt like mattered to me, it could be soul destroying. – Genevieve<br>• Considering the circumstances, I would just say my faith has really helped me a lot through the whole experience and being back at campus with friends and not going through it seemingly alone when you are at home, but with people that are also my same age who are at the same time of life as me, doing the same things as me, that has definitely helped my anxiety most. – Sophia
**Resource Utilization Domain**	
Personnel	• Our school is pretty good about keeping mental health in check, and they have a lot of services for students who are struggling with that kind of business, and so our school is really good about that. Even prior to COVID, they had a ton of hotlines for different issues, and they added a COVID stress hotline. – Doug
Timing and Facility Space	• I had to improvise a lot with using what I had, as opposed to being able to just go to the gym and use a squat rack and stuff like that. So that was a big adjustment for me because I have never had all that taken away from me and had to really think on the fly and stuff like that. – Gina
New Norm	• Once COVID actually hit and things started getting shut down, I had to start buying my own food and cooking at home. I was not necessarily prepared for that; I just did not plan financially. I applied for some of the grants to get some food. Once I got money and I got a job, I was able to cook more regularly and not have to worry about food as much. – Raymond

AIMS Press

© 2022 the Author(s), licensee AIMS Press. This is an open access article distributed under the terms of the Creative Commons Attribution License (http://creativecommons.org/licenses/by/4)
